# Metabolomic profiles associated with bone mineral density in US Caucasian women

**DOI:** 10.1186/s12986-018-0296-5

**Published:** 2018-08-10

**Authors:** Qi Zhao, Hui Shen, Kuan-Jui Su, Ji-Gang Zhang, Qing Tian, Lan-Juan Zhao, Chuan Qiu, Qiang Zhang, Timothy J. Garrett, Jiawang Liu, Hong-Wen Deng

**Affiliations:** 1 Department of Preventive Medicine, College of Medicine, University of Tennessee Health Science Center, 66 N, Memphis, TN 38163 USA; 2 Tulane Center of Bioinformatics and Genomics, Department of Global Biostatistics and Data Science, Tulane University School of Public Health and Tropical Medicine, 1440 Canal St., RM 1619F, New Orleans, LA 70112 USA; 3 Southeast Center for Integrated Metabolomics Core, University of Florida, Gainesville, FL 32610 USA; 4 Medicinal Chemistry Core, Office of Research, University of Tennessee Health Science Center, Memphis, TN 38163 USA; 5 Department of Pharmaceutical Science, College of Pharmacy, University of Tennessee Health Science Center, Memphis, TN 38163 USA; 6 School of Basic Medical Science, Central South University, Changsha, 410013 Hunan China; 7 National Clinical Research Center for Geriatric Diseases, Xiangya Hospital, Central South University, Changsha, 410078 Hunan China

**Keywords:** Bone mineral density, Liquid chromatography-mass spectrometry, Metabolites, Metabolomic, Osteoporosis

## Abstract

**Background:**

Individuals’ peak bone mineral density (BMD) achieved and maintained at ages 20–40 years is the most powerful predictor of low bone mass and osteoporotic fractures later in life. The aim of this study was to identify metabolomic factors associated with peak BMD variation in US Caucasian women.

**Methods:**

A total of 136 women aged 20–40 years, including 65 subjects with low and 71 with high hip BMD, were enrolled. The serum metabolites were assessed using a liquid chromatography-mass spectrometry (LC-MS) method. The partial least-squares discriminant analysis (PLS-DA) method and logistic regression models were used, respectively, to examine the associations of metabolomic profiles and individual metabolites with BMD.

**Results:**

The low and high BMD groups could be differentiated by the detected serum metabolites using PLS-DA (*P*_permutation_ = 0.008). A total of 14 metabolites, including seven amino acids and amino acid derivatives, five lipids (including three bile acids), and two organic acids, were significantly associated with the risk for low BMD. Most of these metabolites are novel in that they have never been linked with BMD in humans earlier. The prediction model including the newly identified metabolites significantly improved the classification of the groups with low and high BMD. The area under the receiver operating characteristic curve without and with metabolites were 0.88 (95% CI: 0.83–0.94) and 0.97 (95% CI: 0.94–0.99), respectively (*P* for the difference = 0.0004).

**Conclusion:**

Metabolomic profiling may improve the risk prediction of osteoporosis among Caucasian women. Our findings also suggest the potential importance of the metabolism of amino acids and bile acids in bone health.

## Background

Osteoporosis is the most common metabolic bone disease, mainly characterized by low bone mineral density (BMD) and deteriorated bone quality/strength, with subsequent increased risk of low trauma osteoporotic fractures. Osteoporosis afflicts over 200 million people worldwide [1]. It has become a serious public health concern due to its climbing prevalence with the population aging and high morbidity, mortality, and health expenditures caused by osteoporotic fractures [2, 3]. Osteoporosis is a multifactorial disorder with several established risk factors, such as aging, rheumatoid arthritis, use of oral glucocorticoids, and menopause in women [4]. Although recent genetic research, mainly genome, transcriptome, epigenome, and even proteome, has greatly enhanced our knowledge in the etiology of osteoporosis, biological mechanisms underlying the development of osteoporosis are still far from being fully understood. Also, the medications for osteoporosis and the prediction tools for osteoporotic fractures remain very limited [5, 6]. Therefore, novel biomarkers for a better understanding of pathogenesis and more powerful prediction/diagnosis/prognosis tools for osteoporosis are still much needed.

Metabolomics is an emerging approach to systematically profile small molecules in biofluids, cells, and tissues [7]. Because metabolites represent the downstream expression of genome, transcriptome, and proteome, their study is hence most powerful to reveal inherent omics variation closest to the disease risk/phenotype [7]. Metabolomics studies have much improved our understanding of some human disorders, such as coronary heart disease, type 2 diabetes, obesity, rheumatic disease, and breast cancer. However, the existing metabolomics studies of osteoporosis in humans are limited to East Asian and European populations, mainly including postmenopausal women [8–11]. A Mendelian randomization study used the genome-phenotype association data from the US population and metabolomics-phenotype association data from the UK and indirectly identified some metabolites associated with BMD [12]. Although promising findings were obtained from these studies, it is still unclear if the findings could be generalized to other race/ethnicity populations since metabolomic profiles have strong genetic determination and are significantly influenced by age and menopause status [13, 14].

It has been established that peak BMD achieved and maintained at ages 20–40 years is the most powerful risk factor for low bone mass and osteoporotic fractures later in life [15, 16]. A 10% increase in peak BMD would delay the onset of osteoporosis by 13 years [15]. In comparison, a 10% increase in the age of menopause, or a 10% reduction in the age-related bone loss would only delay the onset of osteoporosis by 2 years [15]. In this study, we compared the serum metabolomic profiles of young Caucasian women with low and high peak BMD levels with the aim to identify potential early metabolic risk factors/profiles for osteoporosis risk. Identification of such early metabolic risk factors/profiles may effectively and efficiently enable early prevention and intervention for the risk of osteoporosis later in life. To our knowledge, this is the first such metabolomics study (with direct measures of serum metabolites) regarding the osteoporosis phenotype among the US populations.

## Methods

### Study subjects

All the study subjects were from the ongoing Louisiana Osteoporosis Study (LOS) (initiated in 2011), which aims to build a large sample pool and database (with more than 20,000 subjects of various ethnicities in US) for investigating genetic, various omic, and environmental factors for osteoporosis and related health conditions and diseases in Southern Louisiana. The inclusion and exclusion criteria have been described in our previous studies [17, 18]. We used a discordant phenotype design and selected 65 Caucasian women (aged 20–40 years) with low hip BMDs and 71 age-matched Caucasian women with high hip BMDs, respectively, from the bottom and top 20% of the hip BMD Z-score distribution in Caucasian females aged 20–40 years in the LOS. Individuals who were pregnant, had a bilateral oophorectomy, or had any chronic conditions (such as diabetes mellitus, renal failure, liver failure, lung disease, gastrointestinal disease, and inherited bone disease) were excluded from the current study [18].

### Clinical measurements

All participants completed an interviewer-assisted comprehensive questionnaire to collect demographic information, lifestyle (including smoking, drinking, and physical activity), dietary factors (including dairy consumption), reproductive and medical history [17]. In particular, the information on the average number of times of exercise per week and dairy consumption (including milk, yogurt, and cheese) per day were collected using the questionnaire. The serving seizes used were 8 oz for milk and yogurt and 1.5 oz for cheese [19]. Weight was measured in light indoor clothing using a calibrated balanced beam scale, and height was measured using a calibrated stadiometer without shoes. Body mass index (BMI) was calculated as weight (in kilogram) divided by height squared (in centimeters). Waist circumference was measured in the standing position at the midpoint between the lower margin of the least palpable rib and the top of the iliac crest using a stretch-resistant tape. The hip BMD, the combined BMD of the femoral neck, trochanter, and intertrochanteric region, was measured by a dual-energy X-ray absorptiometry machine (Hologic Inc., Bedford, MA) by trained and certified research staff. The machine was calibrated daily, and software and hardware were kept up-to-date during the data collection process. More details on data quality control including the usual covariation for repeated measures have been reported previously [17].

### Metabolomics analysis

The liquid chromatography-mass spectrometry (LC-MS) based metabolomics platform, developed by Dr. Garrett’s lab in the Southeast Center for Integrated Metabolomics at University of Florida was used to perform the metabolomic analysis of the study. The laboratory protocols have been previously described [20]. Briefly, frozen serum samples (− 80 °C) were thawed at room temperature. Each serum sample (100 μL) was mixed with 20 μL internal standard mix [consisting of *myo*-inositol (1,2,3,4,5,6-d_6_), leucine-^13^C_6_, creatine-d_3_ H_2_O (methyl-d_3_), D-leucine-d_10_, L-tryptophan-2,3,3-d_3_, citric acid ^13^C_6_, L-tyrosine ring-^13^C_6_, L-tryptophan ^13^C_11_, L-phenylalanine ring-^13^C_6_, *N*-Boc-L-*tert*-leucine, *N*-Boc-L-aspartic acid, propionic acid ^13^C_3_, succinic acid-2,2,3,3-d_4_, salicylic acid d_6_, caffeine-d_3_ (1-methyl-d_3_), and octanoic acid ^13^C_8_] followed by vortex mixing for 20 s. Next, 800 μL of acetonitrile:acetone:methanol (8:1:1, v: v: v) was added and centrifuged at 20,000×g for 10 min at < 10 °C to remove proteins. The supernatant (250 μL) was transferred to a new 1 mL Eppendorf tube and dried under a gentle stream of nitrogen (Organomation Associates, Berlin, MA, USA). The dried sample was reconstituted in 100 μL of 0.1% formic acid in water containing injection standards mixture (consisting of Boc-L-tyrosine, *N*(alpha)-Boc-L-tryptophan, and Boc-D-phenylalanine) and placed in an ice bath for 10–15 min followed by centrifugation at 20,000×g for 5 min at < 10 °C to remove any debris.

A Thermo Q-Exactive High-Resolution Mass Spectrometer (Thermo Fisher Scientific, Fremont, CA) coupled with a Dionex UHPLC (Dionex Corporation, Sunnyvale, CA) was used to conduct the metabolomic analysis. All samples were analyzed in both positive and negative ion modes with heated electrospray ionization. The mass resolution was 35,000 at m/z 200 with a mass accuracy of less than 5 ppm in positive mode and less than 10 ppm in negative mode. Separation was achieved on an ACE C18-PFP column (100 × 2.1 mm, 2 μm) with 0.1% formic acid in water as mobile phase A and acetonitrile as mobile phase B with a column temperature of 25 °C. The flow rate was 350 μL/min with a total run time of 20 min.

Alignment and feature finding was performed using the open source software MZmine [21]. Metabolite identification was performed by searching an internal retention time library of over 600 compounds. To provide more confident and reproducible study findings, we used a relatively strict criterion, level 1 identification (the most stringent) according to the guidelines of the Metabolomics Standards Initiative (http://www.metabolomics-msi.org), to define the metabolites with known identities in this study. For the level 1 identification, two orthogonal techniques are required for identification of a metabolite. In our experiments, we used retention time of the authentic standard and mass accuracy of 5 ppm or less for positive ions and 10 ppm or less for negative ions to ensure correct reporting of the metabolites. Peaks in the MS were quantified using integrated peak height. Raw area counts for each metabolite in every sample was first normalized to the sum of all injection internal standards to correct for subtle injection differences, and then normalized to the total signal of each sample. The batch correction was performed using well-established Bayes method for microarray data by continually running the neat and pooled quality control samples. Metabolites with missing rates > 20% or coefficients of variation > 20% were excluded from further analyses. Imputation of missing data was performed using the R package ‘missForest’ [22]. The relative abundance data of metabolites were further log transformed and autoscaled to have zero mean and unit variance (z scores) using the R package ‘specmine’.

### Statistical analysis

The characteristics of study subjects were compared between the low and high BMD groups using the *t*-test for continuous variables and the *χ*^*2*^ test for discontinuous variables. To examine the ability of detected metabolites in classifying the low and high BMD groups, we conducted the partial least squares discriminant analysis (PLS-DA) which is a multivariate analysis method and widely used in metabolomics studies [8, 23–25]. PLS-DA is a classification method based on PLS regression, a maximum covariance model of the relationship between *X* (metabolites) and *Y* (BMD groups). It provides a variable importance in projection (VIP) value for each *X* which is calculated as weighted sum of the squared correlation between the PLS-DA components and *Y* and it summarizes the contribution of each *X* to the model [24]. Logistic regression models were also used to examine the associations between individual metabolites and BMD status with the adjustment for potential confounding factors. Since BMI and waist circumference were highly correlated, we only included BMI in the regression models to avoid the problems caused by collinearity. The other covariates included age, age^2^, BMI, current smoking, alcohol drinking, physical activity, and diary consumption. The odds ratios (ORs) associated with one standard deviation increase in the relative abundance of metabolites were calculated. For individual metabolite tests, the false discovery rate (FDR) method was used to adjust for multiple testing. The selection of cutoffs for the VIP score and FDR usually vary by studies, mostly dependent on specific study goals and data structure. In this study, we aimed to select reasonable and comparable numbers of metabolites by these two methods to generate a comprehensive list of BMD-associated metabolites for future replication. In addition, the metabolites which could be identified by both methods might have priorities for further investigation. Because of these considerations, any metabolites with a VIP score ≥ 2.0 in PLS-DA or a false discovery rate (FDR) ≤ 0.2 in the individual metabolite analysis were considered differential metabolites for BMD. To examine the combined effects of differential metabolites on BMD, we constructed multimarker metabolite scores based on identified metabolites by calculating the sum of their z scores (relative abundance) multiplied by the regression coefficients from the logistic regression models. We calculated three metabolite scores using the significant metabolites identified in the PLS-DA, individual metabolite analysis (logistic regressions), and the two methods, respectively. Areas under the receiver-operating characteristic (ROC) curve (AUC) was used to evaluate the performance of the multimarker metabolite scores in discriminating the individuals with low BMD from those with high BMD. All the data analyses were conducted using R packages, including ‘mixOmics’, ‘pROC’, and ‘missForest’. To understand biological functions of significant BMD-related metabolites, a pathway analysis was conducted using the methods implemented in the web server MetaboAnalyst 3.0 [26].

## Results

Table 1 shows the characteristics of the study subjects in the low and high peak BMD groups. The subjects with low BMD (range: 0.61–0.85 g/cm^2^) had a significantly lower level of total hip BMD compared to those with high BMD (range: 0.97–1.37 g/cm^2^) (*P* value < 0.001). The two BMD groups were of a similar age and had no difference in smoking, alcohol drinking, physical activity and dairy intake. However, the subjects with low BMD had significantly lower levels of weight, BMI, and waist circumference compared to those with high BMD.

**Table 1:** Characteristics of the study participants

	Low BMD^a^ (*n* = 65)	High BMD^a^ (*n* = 71)	*P* value
Age, years	31.2 (4.9)	31.8 (5.3)	0.52
Weight, kg	58.4 (7.1)	81.3 (24.1)	< 0.001
Height, cm	163.5 (6.5)	165.6 (6.2)	0.05
BMI, kg/m^2^	21.9 (2.5)	29.7 (8.6)	< 0.001
Waist circumference, cm	71.1 (9.7)	84.6 (18.0)	< 0.001
Current smoking, %	32.3	38.0	0.61
Alcohol drinking, g/day	39.1 (62.1)	34.0 (41.8)	0.58
Physical activity, times/week	3.2 (2.2)	3.1 (2.1)	0.81
Dairy intake, servings/day	1.5 (1.4)	1.6 (1.1)	0.91
Total hip BMD, g/cm^2^	0.77 (0.06)	1.11 (0.08)	< 0.001
Hip BMD Z-score	−1.25 (0.58)	1.63 (0.63)	< 0.001

*BMD* bone mineral density, *BMI* body mass index

^a^Means (standard deviation) for continuous variables and percentages for discontinuous variables

**Fig. 1:**
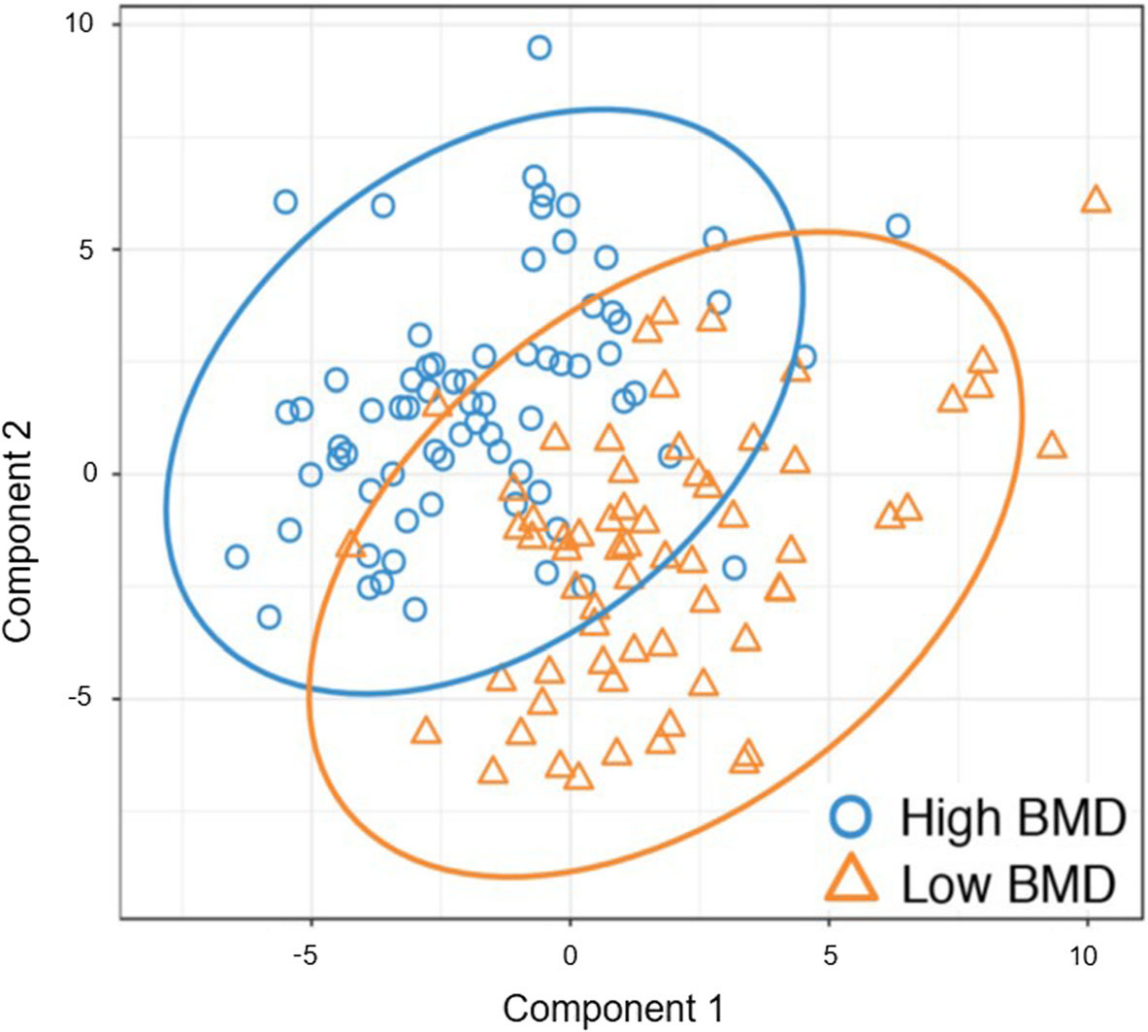
The classification of the low and high BMD groups using PLS-DA. *PLS-DA* partial least-squares discriminant analysis

We identified a total of 192 metabolites with known identities (level 1 identification) and passed quality control using the LC-MS metabolomics platform. In the PLS-DA model, the 192 metabolites could significantly classify the low and high BMD groups (*P* value for 2000 random permutations = 0.008) (Fig. 1). A total of eight metabolites had VIP values > 2.0 and were considered differential for the low and high BMD groups (Table 2).

**Table 2:** The differential metabolites between low and high BMD groups

Metabolite	Class	m/z	RT	VIP score	OR (95%CI)^a^	*P* value
γ-Aminobutanoate	Amino Acid	104.0711	0.81	2.31	0.65 (0.37–1.06)	0.0965
Threonine	Amino Acid	120.0654	1.01	2.44	0.54 (0.31–0.91)	0.0256
L-Cysteine	Amino Acid	122.0267	0.76	1.77	0.44 (0.22–0.79)	0.0115^b^
Taurine	Amino Acid	124.0072	0.72	2.51	1.99 (1.21–3.44)	0.0086^b^
L-Glutamic acid	Amino Acid	146.0458	0.76	1.63	2.18 (1.3–3.94)	0.0055^b^
Stachydrine	Amino Acid Derivative	144.1017	1.23	2.92	0.5 (0.29–0.84)	0.0104^b^
Formylkynurenine	Amino Acid Derivative	237.0865	10.71	1.90	2.51 (1.35–5.16)	0.0065^b^
Isovalerylcarnitine	Lipid	246.1695	8.19	2.30	0.48 (0.26–0.82)	0.0105^b^
Ursodeoxycholic acid	Lipid	357.278	11.53	1.30	2.69 (1.48–5.41)	0.0025^b^
LysoPE (16:0)	Lipid	452.2782	13.42	2.08	1.48 (0.93–2.45)	0.1036
Cholic acid	Lipid	453.2858	11.21	2.20	0.62 (0.36–1.00)	0.0557
Tauroursodeoxycholic acid	Lipid	498.2894	9.99	1.65	2.18 (1.3–3.88)	0.0049^b^
Succinate	Organic Acid	117.0202	2.23	1.75	2.09 (1.23–3.73)	0.0085^b^
N-Acetylneuraminic acid	Organic Acid	308.0989	0.79	2.40	2.15 (1.25–3.98)	0.0092^b^

*CI* Confidence interval, *RT* Retention time, *VIP* Variable importance in projection

^a^Associated with one unit increase in the metabolite

^b^FDR q values ≤0.2

In the individual metabolite analysis, ten metabolites were significantly (FDR q-values ≤0.2) associated with BMD after adjusting for the multiple testing (Table 2). Seven of them (taurine, L-glutamic acid, formylkynurenine, ursodeoxycholic acid (UDCA), tauroursodeoxycholic acid (T-UDCA), succinate, and N-acetylneuraminate) were associated with increased risk for low BMD (ORs > 1.00), and the others (L-cysteine, stachydrine, and isovalerylcarnitine) were associated with decreased risk for low BMD (ORs < 1.00). Four metabolites (taurine, stachydrine, isovalerylcarnitine, and N-acetylneuraminate) were significantly associated with BMD in both the PLS-DA model and individual metabolite analyses (Table 2).

**Fig. 2:**
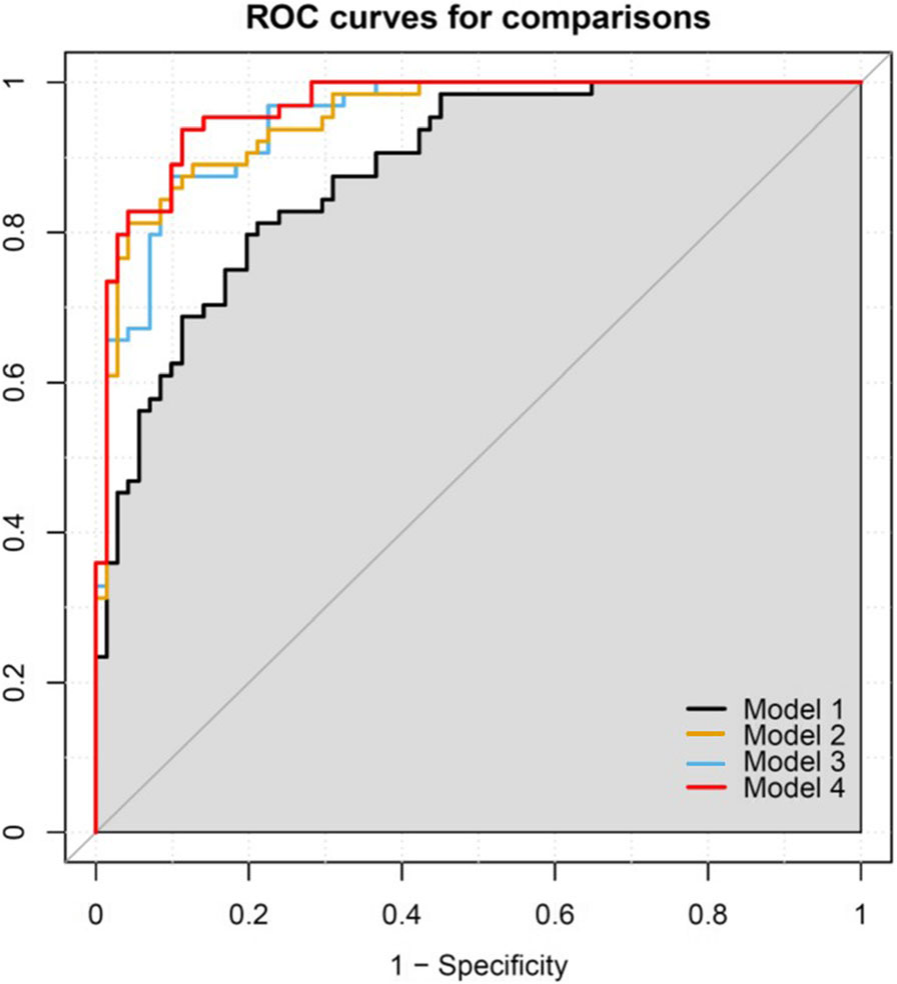
ROC curves of predictive models. *ROC* receiver operating characteristic. Model 1: traditional risk factors including age, age^2^, body mass index, current smoking, alcohol drinking, physical activity, and dairy intake; Model 2: Model 1 + the PLS-DA-derived score generated using γ-aminobutanoate, threonine, taurine, stachydrine, isovalerylcarnitine, lysoPE (16:0), cholic acid, and N-acetylneuraminate; Model 3: Model 1 + individual analysis-derived score generated using L-cysteine, taurine, stachydrine, L-glutamic acid, formylkynurenine, isovalerylcarnitine, ursodeoxycholic acid, tauroursodeoxycholic acid, succinate, and N-acetylneuraminate. Model 4: Model 1 + both methods-derived score generated using all the metabolites identified by the PLS-DA method and individual metabolite analysis

We generated three multimarker metabolite scores using the metabolites identified in the PLS-DA model (PLS-DA-derived score) and in the individual metabolite analysis (individual analysis-derived score) as well as those combined from the two methods (both methods-derived score). All the three metabolite scores were significantly associated with increased risk for low BMD in the logistic regression models with the adjustment for covariates (all *P* values < 0.0001 for the three metabolite scores). We further assessed the extra prediction values of these metabolite scores above and over traditional risk factors (age, BMI, current smoking, alcohol drinking, physical activity, and dairy intake) in discriminating the subjects with low BMD from those with high BMD (Fig. 2). The AUC of the ROC curves from the predictive models with metabolite scores were significantly increased compared with the model including traditional risk factors only, indicating the improved classification of individuals with low and high BMD (Table 3). For example, the AUC of the ROC curve increased about 0.08 by adding the both methods-derived score into the model including traditional risk factors.

**Table 3:** The AUC and comparisons of AUC of the ROC curves from different predictive models

Predictive models	AUC of the ROC curve (95% CI)	Difference of AUC (95% CI)	*P* value for difference
Model 1: traditional risk factors^a^	0.88 (0.83–0.94)	Reference	–
Model 2: Model 1 + PLS-DA-derived score^b^	0.95 (0.92–0.98)	0.07 (0.02–0.11)	0.002
Model 3: Model 1 + Individual analysis-derived score^c^	0.95 (0.92–0.98)	0.07 (0.03–0.11)	0.002
Model 4: Model 1 + Both methods-derived score^d^	0.97 (0.94–0.99)	0.08 (0.04–0.13)	0.0004

*AUC* Area under the curve, *CI* Confidence interval, *PLS-DA* Partial least squares-discriminant analysis, *ROC* Receiver operating characteristic

^a^Including age, age^2^, body mass index, current smoking, alcohol drinking, physical activity, and dairy intake

^b^Generated using γ-aminobutanoate, threonine, taurine, stachydrine, isovalerylcarnitine, lysoPE (16:0), cholic acid, and N-acetylneuraminate

^c^Generated using L-cysteine, taurine, stachydrine, L-glutamic acid, formylkynurenine, isovalerylcarnitine, ursodeoxycholic acid, tauroursodeoxycholic acid, succinate, and N-acetylneuraminate

^d^Generated using all the metabolites identified by the PLS-DA method and individual metabolite analysis

Table 4 shows the seven significant pathways identified in the pathway analysis. The BMD-associated metabolites identified in the current study are mainly involved in the metabolic pathways of amino acids (e.g., the pathways of alanine, aspartate and glutamate metabolism and glycine, serine and threonine metabolism) and lipids (e.g., the pathway of primary bile acid biosynthesis). The amino acids including L-glutamic acid, γ-aminobutanoate (GABA), taurine, L-cysteine, and threonine, and the lipid cholic acid are the major metabolites which match these significant pathways.

**Table 4:** Pathway analysis results using the BMD-associated metabolites

Pathways	Matched Metabolites	Impact	*P* value
Alanine, aspartate and glutamate metabolism	L-Glutamic acid, γ-Aminobutanoate, Succinate	0.2792	0.0001
Butanoate metabolism	L-Glutamic acid, γ-Aminobutanoate, Succinate	0.02841	0.0006
Taurine and hypotaurine metabolism	Taurine, L-Cysteine	0.33094	0.003
Aminoacyl-tRNA biosynthesis	L-Cysteine, L-Glutamic acid, Threonine	0.05634	0.004
Glutathione metabolism	L-Cysteine, L-Glutamic acid	0.01095	0.01
Primary bile acid biosynthesis	Taurine, Cholic acid	0.00849	0.02
Glycine, serine and threonine metabolism	Threonine, L-Cysteine	0.09661	0.02

## Discussion

This, to the best of our knowledge, is the first metabolomics study of BMD conducted directly in Caucasian women of the US. We demonstrated that metabolomic profiles substantially varied among individuals with different risk for osteoporosis. Our study also identified novel metabolites significantly associated with BMD. These novel metabolic markers are promising to improve the risk prediction for osteoporosis. They also highlight the importance of the metabolism of amino acids and bile acids in bone health.

In the present study, we reported for the first time that five amino acids in serum, namely, GABA, threonine, cysteine, taurine, and glutamic acid, were significantly associated with BMD in young and middle-aged Caucasian women. Although in vitro studies have suggested that amino acids might be involved in BMD regulation, it was only until recently that dietary amino acids intake measured by a food frequency questionnaire was reported to be associated with BMD in women and increased intake of alanine and glycine might be beneficial for bone health [27]. Also, recent metabolomics studies reported that the amino acids including glutamine [8], tryptophan [9], and cystine [10] were associated with BMD in postmenopausal women of East Asian. Of these identified BMD-related amino acids in our study, GABA [28], cysteine [29], taurine [30], and glutamic acid [31] have shown some evidence for their potential roles in bone metabolism by in vitro and/or in vivo studies.

Both GABA and glutamate (the anion of glutamic acid) are important neurotransmitters in the central nervous system. However, several lines of evidence show bone cells also express GABA and glutamate receptors and their signaling regulates bone remodeling outside the central nervous system. An in vitro study found that osteoblasts express the GABA_B_ receptor, which negatively regulates osteoblastogenesis through down-regulation of the bone morphogenetic protein-2 expression [28]. However, another study showed that GABA upregulated bone formation genes through activating the GABA_B_ receptor to stimulate osteoblastogenesis in rats [32]. In our study, GABA exhibited a protective effect on BMD with a higher level of GABA associated with a greater level of BMD. Glutamate signaling has been demonstrated to mediate the functional adaptation of the skeleton of mechanical loading [33]. The major type of glutamate receptor, N-methyl-D-aspartame-type glutamate receptor, is widely expressed in osteoblasts, osteocytes, and osteoclasts [31]. Existing evidence supports that glutamate signaling regulates both bone formation and resorption [31]. Our study is in line with a previous metabolomics study which reported that increased glutamine, which can be converted to glutamate in the body, was significantly associated with decreased BMD among Taiwanese women [8].

Cysteine is a non-essential amino acid and is well known to be involved in oxidation-reduction reactions. It is an important source of sulfur in human metabolism. In ovariectomized (OVX) mice, cysteine administration improved BMD and other menopausal symptoms [29]. N-acetyl cysteine, a derivative of cysteine, can function as an osteogenesis-enhancing molecule to accelerate bone regeneration by activating differentiation of osteogenic lineages [34]. Taken together with our study findings, current evidence may suggest that cysteine is a protective factor for osteoporosis.

Taurine supplementation has been linked with increased femur BMD in OVX rats [30]. However, another study did not observe any difference in femur bone mineral content between two groups of OVX rats fed with calcium deficient diet with or without taurine supplementation [35]. This finding might suggest that the effect of taurine on BMD might be dependent on the calcium levels. We found that serum levels of taurine were negatively associated with BMD levels in pre-menopausal women. Therefore, the current findings of the role of taurine in BMD are very preliminary. Future investigations may include examining the effect of taurine on BMD among pre−/peri−/post-menopausal women and its potential interaction with calcium levels.

There is very limited knowledge about the roles of the amino acid threonine and the two amino acid derivatives, stachydrine and formylkynurenine, in bone metabolism. A human study among individuals with spinal cord injury reported that a higher intake of threonine was related to a lower BMD of lumbar vertebrae [36]. However, we found that the serum threonine level was positively associated with BMD among young women. Stachydrine is a derivative of proline, which has shown a beneficial effect on enhancing BMD through increasing serum estradiol and alkaline phosphatase levels and decreasing serum luteinizing hormone in OVX mice [37]. Formylkynurenine is a metabolite of the essential amino acid tryptophan. The kynurenine pathway of the tryptophan metabolism pathway might regulate osteoblastogenesis because oxidation products, such as kynurenine, stopped the proliferation of bone marrow mesenchymal stem cells and inhibited osteoblastic proliferation and differentiation [38]. Further studies are warranted to investigate the mechanisms of these amino acid and amino acid derivatives in bone metabolism.

Some existing evidence indicates the involvement of bile acids in bone metabolism. It is well known that bile acids are critical for the intestinal absorption of lipids and lipid-soluble substances, such as vitamin D. A recent study showed that two types of bile acids had opposite effects on intestinal calcium absorption. Sodium deoxycholate decreased the intestinal Ca^2+^ absorption, while lithocholic acid stimulates the Ca^2+^ absorption. An in vitro study indicated that UCDA, a secondary bile acid which is a metabolic byproduct of intestinal bacteria, could increase differentiation and mineralization of osteoblastic cells [39]. In addition, abnormal bile acid turnover has been linked with osteoporosis in postmenopausal women [40]. Our study found that three bile acids (cholic acid, UDCA, and T-UDCA) in human serum were associated with BMD, further supporting the potentially important role of bile acids in bone health.

In addition to bile acids, another two lipids, isovalerylcarnitine and lysoPE (16:0), were also associated with BMD in our study. Few studies have directly investigated the role of these two lipids in bone metabolism. LysoPE (16:0) is a lysophospholipid (LPL), some subspecies of which have been implicated in cell signaling in bone. For example, lysophosphatidic acid (LPA) is a potent bioactive LPL that mediates osteoblast-osteoclast signaling [41]. The LPA receptor type 1 (PLA1) gene knockout mice had a low bone mass and antagonists of the PLA1 also inhibited osteoclast differentiation [42]. However, a recent study did not observe any beneficial effect for preventing bone loss by pharmacological inhibition of the LPA receptor in mice [43]. The knowledge obtained from these studies are very limited and clarification of the roles of these lipids in bone metabolism are still much needed.

We also identified two organic acids, succinate and N-acetylneuraminic acid, associated with BMD in our study. Succinate is a component of the citric acid cycle, playing an important role in mitochondrial function. Mitochondrial dysfunction has been linked with age-related diseases, including osteoporosis [44]. The major mechanisms include mtDNA damage and mitochondrial-derived reactive oxygen species [44]. N-acetylneuraminic acid is the predominant sialic acid found in mammalian cells. Inconsistent findings have been reported regarding the association between sialic acid and osteoporosis. Quelch et al. observed a lower level of sialic acid in osteoporotic bone compared to controls [45]. However, another study conducted by Mbuyi-Muamba et al. reported an opposite association [46]. Therefore, the links between these two organic acids and osteoporosis need further investigations.

The present study has several important advantages. First, to our knowledge, this is the first such direct metabolomics study about osteoporosis in US Caucasian women. Second, the non-hypothesis-driven untargeted metabolomics approach enabled the study to discover novel metabolites and pathways potentially involved in bone metabolism. Third, we used a discordant phenotype design to maximize the statistical power of identifying metabolites which could distinguish individuals with different risk for osteoporosis. Fourth, multiple variables were adjusted in both individual metabolite and multivariate analyses. Finally, the identification of some metabolites (such as GABA, glutamic acid, and bile acids) with functional evidence in bone regulation by previous in vivo studies, even human studies (such as glutamic acid) enhances the confidence that our findings may present interesting and true metabolites associated with BMD. However, our study does have some limitations. Although the extremely discordant phenotype study design greatly improved the study power, the relatively small sample size might limit the study to detect metabolites with minor effects on BMD. The causal relationship between identified metabolites and BMD could not be inferred because of the cross-sectional study design. In addition, the lack of replication samples from the population with same/similar genetic and environmental background is another limitation which is not negligible but very common for current metabolomics studies. Finally, only metabolites assessed by the LC-MS which had verified chemical identities (level 1 identification) were included in the study, which might have missed some significant metabolites without known identities. However, the metabolites with confident identities will facilitate the interpretation of study findings, such as the pathway analysis, and the potential replication in future studies in different populations. They can also be used to direct functional studies and even translational studies.

## Conclusions

Our study findings suggest that metabolomic changes related to an increased risk for osteoporosis might occur and develop in early life, even before menopausal ages. We have identified novel metabolites in human serum which were significantly associated with the osteoporosis risk among the US Caucasian women. These metabolites highlight the importance of amino acids and bile acids in bone health, providing novel insights into the potential mechanisms of the development of osteoporosis. They also provide potential early biological markers for the risk classification of osteoporosis among healthy women at a relatively young age. Further replications of the study findings among the same and even different populations as well as biological functional studies of the identified metabolites are warranted in the future.

## Abbreviations

AUC: Areas under the curve
BMD: Bone mineral density
BMI: Body mass index
FDR: False discovery rate
GABA: γ-aminobutanoate
LC-MS: Liquid chromatography-mass spectrometry
LOS: Louisiana Osteoporosis Study
LPA: Lysophosphatidic acid
LPA1: LPA receptor type 1
LPL: Lysophospholipid
OR: Odds ratio
OVX: Ovariectomized
PLS-DA: Partial least squares discriminant analysis
ROC: Receiver-operating characteristic
T-UDCA: Tauroursodeoxycholic acid
UDCA: Ursodeoxycholic acid
VIP: Variable importance in projection
